# Treating mid carpal instability conservatively

**DOI:** 10.1186/1753-6561-9-S3-A105

**Published:** 2015-05-19

**Authors:** Carla Bingham

**Affiliations:** 1 Gold Coast Hand Therapy, Queensland, 4216, Australia

## 

Carpal/Wrist instability is the result of a large variety of conditions. Any alteration of the shape of the wrist bones or the connections between them can affect the dynamic balance of forces and the wrists unique multi-plane movements.

Sensorimotor function is a term used to describe sensory, motor and central processes pertaining to joint stability. Wrist joint proprioception and sensorimotor function provides a platform for neuromuscular joint stability.Afferent information from nerve endings affects the neuromuscular control of the wrist joint.

An understanding of these systems as well as the biomechanics of the wrist is critical in its rehabilitation, particularly given the complexity and number of articulations and ligaments within this complex joint.

Mechanoreceptors are sensory end organs reactive to joint pressure, motion and velocity. *Ruffini Ending*, *Pacini Corpuscle* and *Golgi tendon organs* are different mechanoreceptors.

*Ruffini ending* is the predominant mechanoreceptor type found in wrist ligaments. They are slowly adapting, low threshold receptor, which are constantly reactive during joint motion. They react to axial loading and tensile strain in the ligament, but not to ‘weight bearing’ type compressive joint forces. Therefore they signal joint position and rotation.

*The Golgi tendon organ* has only been identified in the large dorsal wrist ligaments, the dorsal radiocarpal and dorsal intercarpal which transvers both the radiocarpal and midcarpal joints. This receptor is important in monitoring tensile strain in the ligament during end range. The DIC ligament is important in maintain stability of the proximal carpal row as well as indirect stability of dorsal midcarpal joint space

*The Pacini Corpuscle* are rapidly adapting, high threshold receptors sensitive to joint acceleration/deceleration and compressive but not tensile forces. Unlike the ankle, where they are found in abundance, they have minor importance in the wrist where they are less prevalent.

The receptors are most pronounced in the dorsal and triquetral wrist ligaments - DRC, DIC, SL, Palmar Lunotriquetral and triquetrocapitate/hamate ligaments.

The arrangements of the wrist ligaments are unique in that the ligaments of the radial column are dense, designed to with stand axial loads given 80% of axial force is transmitted through this side. The dorsal and triquetral ligaments are sensory important structures, and since they traverse both the radiocarpal and midcarpal joints signal throughout wrist joint motion.

Proprioceptive reflexes between wrist ligaments and forearm muscles have recently been demonstrated particularly in the antagonist muscles in each wrist position, indicating a joints protective function. Ongoing global stability of the wrist joint is evident in later reactions, which initiate simultaneous co-activation of the wrist flexors and extensors. This delicate balance of co-contraction is also believed important in maintaining smooth and even joint motions.

After a wrist injury or wrist surgery, most patients have been immobilized during the posttraumatic or postoperative period. Therefore all of the afferent kinesthetic information from the wrist joint, the skin, and muscle spindles has been lost. Complete with the reduced visual feedback a patient’s total conscious awareness is diminished. Furthermore after ligament injury periarticular muscles are frequently weak, which will result in a neuromuscular imbalance during concentric contractions.

## Our wrist neuromuscular rehabilitation programme includes: -

• Early sensorimotor feedback including mirror therapy.

• Dynamic stabilization to compensate for compromised ligaments

• Promotion of motion/strength in muscles that are joint protective.

• Re-establish the kinematic balance between muscles, ligaments and their mechanoreceptors.

• Reactive muscle activation exercises.

• Isometric exercises

• Eccentric exercise patterns with a focus of the co-activation stabilising forces of the antagonist muscle.

• Co-activation exercises, simultaneous contraction of agonist and antagonist muscles, to promote global wrist stability a balanced wrist motion.

• Reestablishing unconscious activation of muscles by restoring the neuromuscular reflex pattern or reactive muscle activation (RMA).

• Incorporation of nerve gliding with dynamic stabilisation.

## This type of programme has brought a new wave of strengthening and rehabilitation of the wrist and includes the use of unconventional exercise tools such as: -

• Slosh pipes

• Powerball/Rollerball (gyroscope)

• Weighted balls

• Flex bars

• Sand bags

• Mirror therapy

• Interx therapy incorporated into exercise regime

I will cover the specific types of exercises that address the scientific basis of this programme that have been found to be clinically successful and outline the scope of poor performance and pitfalls.

